# The environmental impact of diagnostic imaging: opportunities for pediatric radiologists

**DOI:** 10.1007/s00247-026-06656-5

**Published:** 2026-05-22

**Authors:** Jonathan Gross, Sumit Pruthi, Reed A. Omary, Elizabeth J. Snyder

**Affiliations:** 1 Cohen Children’s Medical Center, New Hyde Park, USA; 2 Baylor College of Medicine, Houston, USA; 3 Department of Radiology and Radiological Sciences, Vanderbilt University Medical Center, 1211 Medical Center Dr, Nashville, TN 37232 USA

**Keywords:** Child, Climate change, Diagnostic imaging, Environment, Radiologists

## Abstract

Healthcare operations are a major contributor to human-produced greenhouse gas emissions worldwide and in the USA in particular. Medical imaging, largely due to its high energy use requirements, disproportionately contributes to healthcare’s negative environmental impacts. Although there has been growing concern about radiology’s role in climate change, most existing knowledge of radiology’s impact derives from adult radiology departments. Pediatric radiologists, however, have compelling reasons to consider and work to reduce our specialty’s contributions to climate change: our pediatric patients are uniquely vulnerable to the negative effects of climate change and face wide-ranging impacts to their overall health and well-being. The aim of this manuscript is to review the major environmental impacts from radiology and to highlight potential areas for improvement.

## Introduction

Climate change, primarily due to human-produced greenhouse gas emissions (GHGs), has wide-ranging adverse effects on the Earth’s environment and, as a result, negatively impacts the health of patients. Although climate change threatens nearly all parts of society, its negative effects disproportionately impact children and other vulnerable populations [1].

Such effects impact children’s health and well-being in both direct and indirect ways. Direct effects include the negative impacts of altered weather patterns which can be wide ranging [1]. For example, more frequent and worsening heatwaves increase morbidity and overall mortality particularly for infants and young children [2]. Increased frequency and severity of floods can result in increased rates of drownings and injuries [3]. Increased wildfires can worsen respiratory health and increase the risk of infectious disease [1, 4].

Indirect effects of climate change on children’s health are similarly wide ranging. Rising temperatures are associated with the increased rates of several infectious diseases such as vector-born illnesses like malaria and dengue [1]. Worsening air pollution, a result of multifactorial causes including GHG and toxin emissions as well as increased droughts and wildfires may also worsen respiratory health [1, 5]. Altered weather patterns may increase rates of food and water insecurity as well as malnutrition, to which children are uniquely vulnerable. Finally, more remote indirect effects may include increased rates of childhood poverty which can have a long-term negative impact on a child’s health and well-being [6]. Given these risks, the American Academy of Pediatrics recently stated that failure to take “prompt, substantive action” against climate change “would be an act of injustice to all children” [7].

Healthcare itself contributes 4.4% of global GHGs and 8.5% of US GHGs, with radiology contributing a disproportionate amount given its substantial carbon footprint [8–11]. Indeed, medical imaging alone is estimated to contribute 1% of all global GHG emissions [12]. It is imperative, therefore, for pediatric radiologists to understand the environmental impacts of medical imaging and strive for ways to reduce its effects to protect the health of our planet and our patients. The aim of this review is to provide an overview of the largest drivers of negative environmental impacts of radiology and discuss potential strategies to reduce them.

## Measuring environmental impacts of radiology

Increasing attention has been paid to radiology’s contribution to planetary health in recent years and several methods have been used to quantify its effects [13]. Accurate measurements are essential to understand the scope of its environmental impact, to develop evidence-based interventions, and to subsequently evaluate the effectiveness of such interventions. The most common methods to evaluate radiology’s environmental impacts are detailed below. Importantly, although some types of analyses are quite comprehensive, they can still vary in scope depending on how the analyses are performed, and geographic differences, such as components of local electricity grids, can lead to variations in GHG emissions [13, 14].

### Life cycle analysis

Life cycle assessment (LCA) is a comprehensive method to evaluate the total environmental impacts of a process or a product throughout its lifecycle. LCAs begin with resource extraction, continue to manufacturing and distribution, extend to the product’s actual use, and end with disposal. In addition to calculating the GHGs of a product or process, LCA may also evaluate other environmental impacts, including contributions to water use, ozone depletion, eutrophication, or the creation of carcinogens. For example, an LCA of MRI use in a radiology department might include not only the energy consumption of the machines but could also include the environmental impact of production and distribution of the machines, the production of intravenous contrast, the production and laundering of linens, and the energy use of radiology workstations and data storage [15]. Relatively few LCA studies have been conducted of imaging departments, limiting our ability to capture radiology’s full environmental impact [13].

### Carbon footprint assessment

Evaluating the carbon footprint of a device or process focuses on estimating GHGs and may evaluate both direct and indirect causes of emissions, from manufacturing to electricity use to the generation of waste [11]. Unlike with LCA, carbon footprint assessments focus only on GHG emissions, rather than on other environmental impacts. Such analyses, however, can still be quite comprehensive, as substantial environmental impacts in radiology are caused by direct GHGs, such as energy use from machines and equipment, patient transport, and heating, lighting, and cooling systems [11].

### Energy consumption analysis

Energy consumption analysis focuses specifically on the electricity used by radiology equipment. Most prior studies in radiology have focused on the energy consumption of radiology devices, such as imaging machines and/or diagnostic workstations during their operation, often through direct measurement such as through power meters or current sensors [13, 16–18]. While energy consumption is a major factor in the environmental impact of radiology, it is less comprehensive than carbon footprint assessment or a detailed LCA.

### Waste auditing

Waste auditing quantifies all types of waste generated from a given process. It commonly includes packing materials, single-use supplies, and other waste, such as pathological or radioactive waste [19]. This information can help identify potential ways to reduce or recycle waste. Waste analysis may be particularly useful to evaluate interventional radiology procedures, where single-use supplies are common [19–22].

## Environmental impacts of imaging modalities

**Fig. 1:**
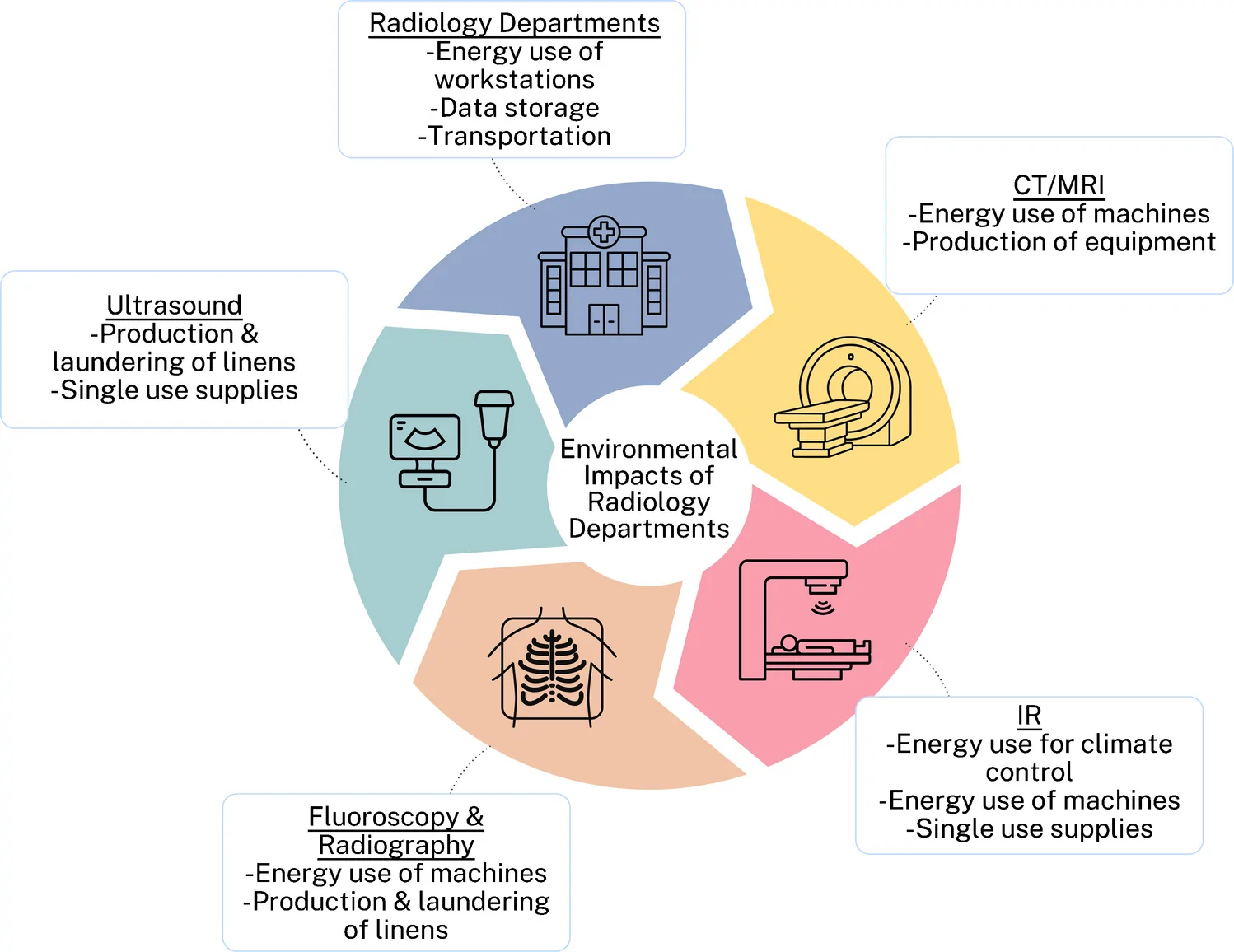
Major sources of negative environmental impacts in radiology departments Abbreviations: Computed tomography (CT), magnetic resonance imaging (MRI), interventional radiology (IR)

**Fig. 2:**
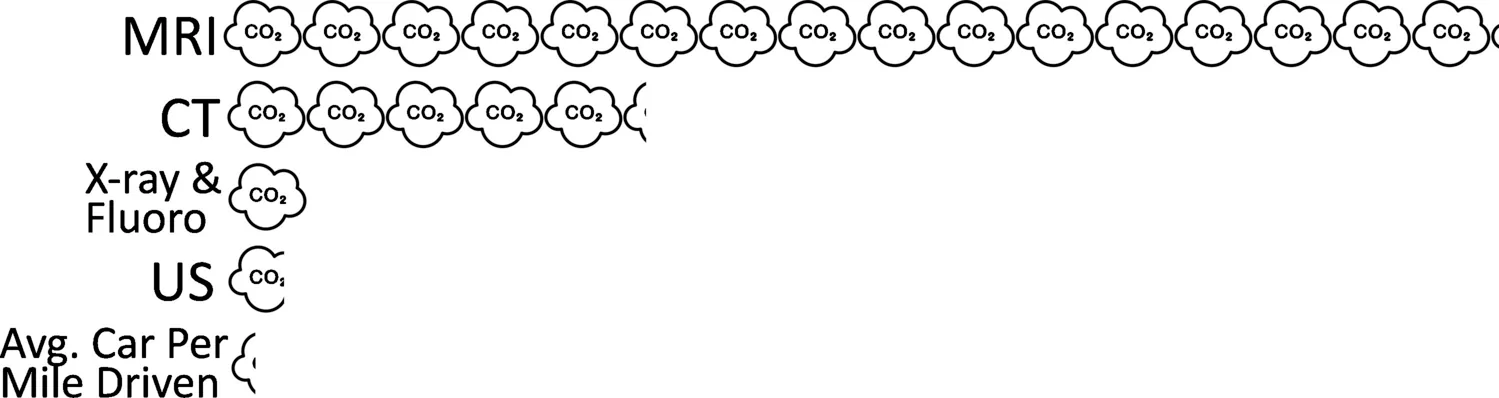
Average CO_2_ emissions per scan for each modality compared to the average CO_2_ emissions for a passenger car per mile driven [9, 14]. Abbreviations: Magnetic resonance imaging (MRI), computed tomography (CT), fluoroscopy (fluoro), ultrasound (US), Average (Avg.)

Currently, nearly all our knowledge of the environmental impacts of radiology is from adult imaging departments [13]. Given similarities in equipment usage and processes, general themes of the environmental impacts of each modality are likely similar between both adult and pediatric imaging departments (Figs. 1 and 2).

### Magnetic Resonance Imaging/Computed Tomography 

Magnetic resonance imaging (MRI) by far contributes to the most GHG emissions of any imaging modality, followed by computed tomography (CT) [13, 14]. For example, an average MRI machine emits 51.5 metric tons (MT) carbon dioxide emissions (CO2e) per year from energy consumption alone, while an average CT machine emits 11.8 MT CO2e per year from energy consumption (much higher than the average X-ray or ultrasound (US) machine which emit 0.7 and 0.3 MT CO2e per year, respectively [13]. Such impacts are mostly from daily operational electricity use [14–16]. In a recent evaluation of an adult imaging department, energy use alone from MRI and CT scanners contributed to 38% and 10%, respectively, of the department’s overall emissions [14]. Importantly, these machines consume significant energy even when not in active scanning mode. In fact, the energy used during “ready-to-scan” but unoccupied modes contribute more to their overall energy consumption compared to active scanning modes [15]. In one study, MRI machines consumed up to 75% of their energy in low-power or “ready-to-scan” mode while only 25% of its energy consumption occurred during active scanning [15]. Similarly, up to 77% of a CT scanner’s energy consumption can occur from low-power or “ready-to-scan” mode [15]. Although CT and MRI still consume energy in stand-by or off-modes, switching into these modes has been shown to offer substantial energy savings [13, 23]. For example, simply shutting down an MRI machine overnight could reduce energy use by the scanner by 25–33% [15, 23].

Energy consumption and emissions from manufacturing MRI and CT scanners also contribute to their environmental impacts, much more so than the production of X-ray and ultrasound machines [14, 24]. Specifically, the manufacturing of MRI and CT machines contributes to 10% and 17% of their emissions, respectively [15]. In addition to energy consumption, the production of CT and MRI scanners also contributes to negative environmental impacts in the domains of carcinogenics and ecotoxicity [15].

Significant environmental costs MRI and CT imaging also stem from the lifecycle of disposable items used, such as positioning devices, gloves, IV starter kits, slippers, linens, and contrast agents [15]. Indeed, disposable supplies have been found to contribute 26% and 16% of all an MRI and CT suite’s GHG emissions, respectively [15]. Importantly, these single-use items used for CT and MRI scans contribute not only to GHG emissions but also to effects such as ozone and fossil fuel depletion [15].

Contrast materials carry their own environmental concerns. Iodinated contrast agents frequently enter wastewater and persist because they resist standard treatment processes [25]. Gadolinium has also been found in tap water and carbonated drinks [26]. Accumulation of contrast media in natural water sources can generate harmful by-products, posing ecological risks (e.g., toxicity, acidification, eutrophication) and potential human health effects [25, 26].

### Fluoroscopy and radiography

Although radiology and fluoroscopy are some of the most widely used imaging modalities worldwide, particularly in pediatric radiology, less is known about their environmental impacts compared to other modalities [9, 14, 27]. While their overall impacts are less than that of CT and MRI, the effects of fluoroscopy and radiography are not negligible. In an adult radiology department, for example, radiography and fluoroscopy still accounted for 12% (0.55 kt CO2e over a 10-year period) of the department’s entire GHG emissions [14]. Although similar data are not available for pediatric radiology centers, given the frequent use of X-rays in pediatric patients, such studies likely contribute to a similar, if not greater, proportion of GHG emissions in pediatric radiology.

Energy use from radiology and fluoroscopy machines is the largest driver of their GHGs, similar to CT and MRI. While energy consumption when not in active scanning mode is a problem for all modalities, fluoroscopy machines in particular may spend a high proportion of time in low-power mode as most fluoroscopic studies are performed during routine business hours [28]. In a recent evaluation of scanner use in an adult radiology department, fluoroscopy scanners spent up to 92% of their time in nonscanning mode, during which time they consume more than 90% of their total power consumption [14, 27].

Nearly a quarter of all emissions caused by radiography and fluoroscopy services come from the production and laundering of linens, with laundering contributing approximately double the amount of emissions compared to production [27]. Flat sheets that may be used to cover the scanner beds for each patient and are laundered after every use contribute the largest to these effects. The production of both scanners and linens also contributes to other negative environmental effects outside of GHGs, including emissions of ozone depletion compounds, acidification, smog, and carcinogenics [14, 27].

### Ultrasound

Other than radiographs, ultrasound is likely the second most common imaging modality in pediatric patients [29, 30]. Ultrasound contributes to overall less GHGs compared to MRI, CT, and even radiography [13, 14, 18, 31]. Specifically, mean carbon emissions per US scan were estimated to be 1.1 kg CO2e, compared to 14.7 kg CO2e for MRI and 1.4 kg CO2e [27].

In contrast to other modalities, the energy use of ultrasound machines is overall a minor driver of its environmental impact. Instead, the production and laundering of linens and the use of single-use supplies are much more significant causes of GHG emissions, contributing to approximately 35% and 34% of the modality’s overall emissions [31]. The single-use supplies that most contribute to ultrasound’s environmental impact include gel, gloves, and probe covers [31]. In addition to contributing to GHGs, single-use supplies also have other negative environmental impacts, including ozone depletion, non-carcinogenic emissions, and ecotoxicity, mostly from their production [31]. For example, the processes used in the production of terephthalic acid—a precursor material used to create various textiles and plastic packaging which are used in nearly every single-use supply item used in ultrasound—often generate other ozone-depleting compounds.

### Interventional radiology

Like diagnostic radiology, interventional radiology (IR) is a resource-intensive specialty which contributes to climate change. An LCA found that over just 5 days, one hospital’s IR practice emitted 23,500 kg CO2e, equivalent to emissions generated by driving 59,000 miles (for reference, the average American drives approximately 13,500 miles per year) [20]. The primary sources of emissions were the energy used to power climate control systems, energy used to power imaging equipment, and emissions from manufacturing and transporting single-use surgical supplies.

Like CTs and MRs, angiography machines consume large amounts of energy even when they are not in use. One study found that 76% of the energy used to power an IR angiography unit was consumed while the machine was in idle or stand-by mode, highlighting the wastefulness of current practices [17].

Single-use surgical supplies alone generate approximately 40% of an IR department’s GHG emissions [20]. Many of the supplies that are opened in the OR and IR suite go unused and contribute to environmental harm needlessly [32–34].

## Other sources of environmental harm in radiology departments

In addition to the energy use and manufacturing of imaging machines and the supplies required to obtain imaging studies, several other aspects of pediatric radiology contribute its environmental impact.

The energy use of diagnostic workstations and the production and energy use required for data storage devices also contribute to radiology’s environmental impact, though their effects are lower than that of most diagnostic imaging equipment, particularly CT and MRI [14, 31]. For example, in an adult radiology department, workstations and data storage contributed to 12% of the department’s GHGs (0.57 kt CO2e over a 10-year period), compared to 48% from MRI and 24% from CT [14]. Like imaging machines, diagnostic workstations can consume substantial energy even when not in active [35]use; in one study, shutting down devices including PACS workstations, printers, and monitors saved 9.26 MT CO2e per year [35].

One additional aspect of pediatric radiology that may contribute to its environmental footprint is the potential need for anesthesia, especially for MRI exams. Although the direct contribution of anesthesia use in pediatric MRI has not been studied, an estimated 25–45% of children require anesthesia for MRI exams, although attempts have been made to decrease this rate in recent years [36–38]. Inhaled anesthetics, specifically desflurane and nitrous oxide, commonly used for pediatric sedation, are much more potent GHGs than carbon dioxide and have been shown to contribute to up to 7% of a children’s hospital’s GHGs [39].

Clinical use of artificial intelligence (AI) models is relatively new and rapidly advancing. Thus, less is known about its overall contribution to radiology’s environmental impact. Nevertheless, some AI applications, particularly large language models (LLMs), are known to require a high amount of energy for use [40]. Although there is no data on the use of AI models in radiology specifically, the emissions just from the training phase of one LLM, GPT-4, were estimated to range from 12,456 tons to 14,994 tons of CO2e [41]. Unfortunately, the energy used in the inference stage of LLMs scales with its use and can therefore far exceed that of the training phase [40, 41]. For example, for GPT-3, given the number of estimated daily users, the electricity consumed in the inference phase would match that of the training phase in less than 2.3 days [41]. Despite the energy consumption required by LLMs, efforts can still be made to mitigate the environmental harms of these systems by actions such as using fewer or more energy-efficient central processing units, compressing stored data, and reducing the use and development of redundant AI models that perform similar tasks [40].

Conversely, AI may also be leveraged as a tool to reduce the environmental impacts of imaging departments. For example, AI-based programs can significantly reduce MRI scan times and streamline patient scheduling to reduce idle scanner time, and clinical decision support tools could aid in the reduction of unnecessary or low-value imaging [40]. Further study is needed as these tools are developed and implemented to accurately delineate their value and overall environmental impact.

Little is known about the contribution from employee and patient transportation to imaging sites to radiology’s overall environmental impact in both the pediatric and adult populations. Patient transportation, however, has been estimated to contribute to about 6.4% of the GHGs from healthcare delivery in the USA [42]. In a study of the environmental impact of an adult nuclear medicine department in France, 67% of emissions were estimated to be from patient and staff travel [43]. Given that pediatric patients may require care at specialized centers, they may need to travel long distances for care, especially those who live in rural communities [44]. Although not directly related to the delivery of patient care, the impact of travel to professional conferences and job interviews for radiologists and staff could also be considered within radiology’s footprint [45]. A recent estimate of the airplane-related carbon emissions from an RSNA meeting was 39.5 million kg CO2e [45].

## Opportunities for reduction

Substantial opportunities exist to reduce the environmental effects of pediatric radiology departments. Notably, we can apply some of the unique aspects of pediatric radiology to drive change in our departments. Furthermore, adult radiology departments could learn from common principles used in pediatric radiology to reduce the impact of their imaging practices as well. For instance, pediatric radiologists’ close relationships with referring providers and our experience with reducing children’s exposure to radiation could be leveraged to promote the use of lower-impact modalities, such as ultrasound, when feasible. Our drive to reduce the use of sedation and intravenous contrast can be used to help develop interventions that also reduce environmental harms from imaging, such as using lower-impact modalities or using the shortest possible protocols to answer a specific clinical question.

Pediatric radiologists are also uniquely positioned to focus on reducing the environmental harms of imaging because of their role as advocates for the overall health and well-being of children [46]. Because children are uniquely vulnerable to the impacts of climate change, planetary health can be viewed as a public health and health equity issue. As such, pediatric radiologists have a valuable role to play in advocating for climate change mitigation strategies.

Many potential interventions apply to all modalities or entire radiology departments. Pediatric radiologists should continue to strive to reduce unnecessary or low-value imaging and use the least energy intensive modality as possible to still answer the clinical question (Table 1). Clinical decision support tools, including those which take environmental impacts into consideration, would also be useful. Techniques to reduce the use of intravenous contrast, such as the implementation of multi-use vials, maintaining a diverse stock of single-use vials, or using syringeless contrast injectors may reduce significantly reduce waste and have been implemented successfully, albeit at a small scale, within radiology departments [47]. Several institutions also participate in iodine recycling programs offered by contrast manufacturers.

**Table 1:** Major opportunities to reduce pediatric radiology’s environmental impact

General goal	Specific interventions
Reduce unnecessary or low-value imaging	• Improve clinical decision support • Further develop appropriate use criteria
Use lowest energy intensive modality possible	• Include environmental impacts in clinical decision support tools
Reduce energy use of machines	• Turn off machines overnight if not in use • Improve scheduling and patient throughput processes • Collaborate with vendors to develop scanners that can quickly shift into low-power mode or off-modes • Collaborate with utility companies and local governments to incorporate renewable energy sources into local electrical grids • Develop and implement renewable energy microgrid
Adopt circularity processes for manufacturing and replacement	• Upgrading or refurbishing equipment rather than replacing • Switching to reusable supplies rather than single use
Reduce use and impact of supplies	• Reduce dose and unnecessary use of intravenous contrast • Ensure appropriate use of linens • Improve protocols to reduce need for anesthesia • Reduce or phase out desflurane and nitrous oxide for sedation in favor of lower-impact alternatives like sevoflurane • Switch to reusable supplies rather than single use • Hone storage and stocking practices • Create different pre-assembled equipment trays for procedures

As scanners can consume substantial energy even when not in scanning mode, focusing on ways to reduce their energy while not in use may have substantial impact [14]. For example, shutting off IR machines at night and on weekends may save over 4 metric tons of CO2e per angiography unit annually [17]. At one institution, powering down MRI machines overnight significantly reduced power consumption, which could reduce emissions by 8.7–14.9 metric tons CO2e per year [23]. Departments can also work with vendors to develop scanners that can more quickly shift into low-power mode or off-modes [12, 16]. Improving scheduling and patient turnover procedures is another way to reduce energy use. Additionally, departments can consider lifetime energy usages from vendors when making purchasing decisions [12].

Energy savings can be realized by powering down idle devices, such as workstations and printers, when not in use [35]. Data storage practices can also be scrutinized; tiered data storage systems can be considered—while regularly accessed data could be stored on faster storage devices, slower, more energy-efficient devices could be used to store data that is not frequently used [40]. Health systems can also ensure they are using appropriate data compression techniques.

In IR suites, climate settings should be allowed to drift when rooms are not in use. While temperature, humidity, and air exchanges must be maintained within tight parameters while IR suites are occupied, climate settings are permitted to drift while rooms are empty. Currently, most practices do not set back climate settings when rooms are unoccupied, which leads to substantial waste of energy [20]. By installing occupancy sensors and automatically setting back climate control settings, operating rooms have decreased energy consumption by up to 70% without increasing infection risk [48, 49]. The investment needed to retrofit equipment can pay for itself quickly in energy savings [49].

If sedation is required for imaging or procedures, using limiting the use of desflurane and nitrous oxide would be impactful [39]. Indeed, a recent sustainability quality improvement project performed in a children’s hospital resulted in an 87% reduction in GHG emissions from inhaled anesthesia used in the operating rooms over a 5-year period [39].

To reduce the environmental impact of surgical supplies, practices should strive to use less. For IR procedures, creating different pre-assembled equipment trays for procedures of different complexity, developing physician-specific equipment preference cards for commonly performed procedures, and fostering a culture in which supplies are opened only if needed can reduce waste [50–52]. Minimizing redundant inventory and preferentially using equipment that is near expiration decrease the likelihood that supplies will expire before being used and has been shown to reduce waste in real-world conditions [53]. Switching from disposable personal protective equipment and surgical supplies to equipment that can be reprocessed and reused can decrease emissions, especially in areas where electricity is sourced primarily from renewables and natural gas [51, 52].

Other opportunities to reduce radiology’s environmental impact include (a) advocating for a cleaner energy grid and (b) switching from linear to circular replacement of imaging equipment. Circular approaches minimize the use of primary materials by reusing, recycling, and refurbishing parts of existing scanners that are already in use [54].

While there are numerous potential areas to reduce the environmental impact of medical imaging, it must be acknowledged that barriers do exist to the implementation of some strategies. Some interventions, such as those requiring collaboration with policy makers, local utility companies, or large vendors, require institutional support, which may be lacking. Lack of funding and leadership support and insufficient time are also real barriers that may limit individual radiologists’ ability to implement some of these changes [55].

## Conclusion

Given the vulnerability of our patient population to the effects of climate change, we call on pediatric radiologists to reduce the environmental impact caused by our specialty. We also recommend that our specialty conduct more sustainability research focused on the pediatric population, recognizing the timeless dictum that children are not just little adults. Through further research, evidence-based interventions, and advocacy, we can reduce the environmental harms of imaging, while protecting the health and well-being of children.

## Data Availability

No datasets were generated or analysed during the current study.
